# Wythe’s Pocket Dose Book

**Published:** 1853-01

**Authors:** 


### WYTHE'S POCKET DOSE BOOK.

This is one of those cheap little manuals offered to the physician of which there are so many in our day. They are exceedingly convenient and useful to the practitioner whose business calls him far away from home and who cannot consult the authors in his library in every emergency. Such a manual as this —a *dose* and *symptom* book—which he could carry in his pocket would be most acceptable to the physician many times when in doubt. The contents are arranged alphabetically for convenient reference. It is offered to the profession by Lindsay & Blakiston, who, no doubt, will find ready sale for it.

